# Efficacy, safety and policy implications of anti-amyloid monoclonal antibodies for Alzheimer’s disease: protocol for a living systematic review and meta-analysis

**DOI:** 10.1192/bjo.2026.12044

**Published:** 2026-07-09

**Authors:** Saehyeon Kim, Haruhiko Oda, Rie Oyama, Rei Makishi, Sohail Bade, Sahil Bade, Irshad Ally, Yuka Iijima, Yuki Gibo, Kota Minami, Fumitoshi Fukuzawa, Shohei Sanji, Kentaro Inamine, Hironori Kishi, Sunjun Huh, Takaki Tanifuji, Mone Nagahiro, Norio Watanabe

**Affiliations:** Department of Medicine, SUNY Upstate Medical University, USA; Hyogo Mental Health Centre, Hyogo, Japan; General Internal Medicine, Saint Marianna University School of Medicine Hospital, Japan; Faculty of Medicine, University of the Ryukyus, Japan; Department of Family Medicine, SUNY Upstate Medical University, USA; Biomedical Informatics, University at Buffalo Jacobs School of Medicine and Biomedical Sciences, USA; Johns Hopkins Bloomberg School of Public Health, Johns Hopkins University, USA; Medical Training Centre, Saga University Hospital, Saga, Japan; Morningside/West, Icahn School of Medicine at Mount Sinai, USA; Department of Psychiatry, National Centre Hospital, National Centre of Neurology and Psychiatry, Kodaira, Japan; Shizuoka General Hospital, Shizuoka, Japan; Department of Pharmacology, UT Health San Antonio, The University of Texas at San Antonio, USA; Department of Psychiatry, Soseikai General Hospital, Kyoto, Japan

**Keywords:** Alzheimer’s disease, monoclonal antibodies, amyloid-β, disease-modifying therapy, systematic review

## Abstract

**Background:**

Dementia affects approximately 6–13% of adults aged 65 years and older, with Alzheimer’s disease accounting for most cases. Established symptomatic therapies, including acetylcholinesterase inhibitors and memantine, provide limited benefit and do not modify disease progression. Multiple monoclonal antibodies (mABs) targeting different amyloid-β species have been developed as potential disease-modifying therapies; because some agents have entered clinical use whereas others remain investigational, a continuously updated synthesis of their efficacy and safety is needed.

**Aims:**

To evaluate the efficacy and safety of all anti-amyloid mABs for adults with Alzheimer’s disease, using a living systematic review and meta-analysis.

**Method:**

We will conduct a living systematic review and meta-analysis in accordance with the Cochrane Handbook, Preferred Reporting Items for Systematic reviews and Meta-Analyses (PRISMA) 2020 and the PRISMA extension for living systematic reviews. Randomised controlled trials comparing any approved or investigational anti-amyloid mAB with placebo, standard care or active comparators will be included. Searches of Ovid MEDLINE, Embase, Cochrane Central Register of Controlled Trials, ClinicalTrials.gov and WHO International Clinical Trials Registry Platform will be updated every 6 months. Meta-analyses will be conducted separately for each antibody molecule using random-effects models. Critical outcomes include global clinical change and disease severity, cognitive abilities, functional ability and dependency, and safety (serious adverse events, treatment discontinuation and amyloid-related imaging abnormalities). Important outcomes include neuropsychiatric symptoms, quality of life and health system outcomes. Certainty of evidence will be assessed using the methodology Grading of Recommendations, Assessment, Development and Evaluation.

**Results:**

This article describes a protocol; therefore, no review findings are available at this stage.

**Conclusions:**

This living systematic review will provide an up-to-date synthesis of the benefits and harms of anti-amyloid monoclonal antibodies to inform clinical decision-making and health-system planning in Alzheimer’s disease.

The global prevalence of dementia among adults aged 65 and older is estimated to range from 6 to 13%, with Alzheimer’s disease accounting for the majority of cases.^1,2^ Alzheimer’s disease is a progressive and fatal neurodegenerative disorder that leads to cognitive and functional decline, loss of independence and increased mortality. Diagnosis remains primarily clinical, supported by neuropsychological assessment and, increasingly, by biomarkers such as amyloid positron emission tomography (PET), magnetic resonance imaging (MRI) and cerebrospinal fluid analysis for amyloid and tau proteins.^3^

For nearly three decades, standard therapy has relied on acetylcholinesterase inhibitors and memantine, which offer modest symptomatic relief but do not alter the course of the disease.^4^ More recently, monoclonal antibodies (mAbs) targeting amyloid-β have emerged as potential disease-modifying therapies.^5^ These antibodies vary in their mechanisms, binding to distinct amyloid-β conformations ranging from soluble monomers to aggregated protofibrils, fibrils and plaques.

Early mAbs such as solanezumab, bapineuzumab and crenezumab failed to show convincing clinical benefit despite reducing certain amyloid species.^6–9^ More recently, next-generation antibodies including aducanumab, lecanemab and donanemab have demonstrated greater amyloid clearance and modest cognitive benefits in selected populations.^5,10–13^ Although aducanumab was later withdrawn due to uncertain efficacy and safety, the advent of these therapies represents a pivotal shift in the therapeutic landscape of Alzheimer’s disease.

Given the rapid development of additional amyloid-targeting antibodies – spanning both investigational and approved agents – a comprehensive and continuously updated synthesis of evidence is required. This living systematic review aims to include all anti-amyloid mABs developed for the treatment of Alzheimer’s disease, irrespective of their regulatory status or generation, and will be continuously updated as new data emerge.

## Method

This review will adhere to the Cochrane Handbook for Systematic Reviews of Interventions and will follow the Preferred Reporting Items for Systematic reviews and Meta-Analyses (PRISMA) 2020 guidelines for transparent reporting, together with the PRISMA extension for Living Systematic Reviews (PRISMA-LSR 2024).^14,15^ This systematic review protocol was prospectively registered in PROSPERO (registration no. CRD420251163998). The detailed registration record can be accessed online (https://www.crd.york.ac.uk/PROSPERO/view/CRD420251163998).

Further details regarding the living systematic review processes are described below.

### Types of studies

We plan to include randomised controlled trials (RCTs) involving participants diagnosed with Alzheimer’s disease, in which anti-amyloid mABs were investigated.

To maintain methodological robustness, we will exclude cluster-randomised, quasi-randomised and observational designs. Both published and unpublished trials will be eligible, with no restrictions regarding language or publication status.

Open-label extension data will not be included in quantitative synthesis because of the absence of randomisation and control groups, and on account of the high risk of selection and survivor bias.

### Types of participants

We will include adults diagnosed with Alzheimer’s disease, with a focus on those at the stage of early symptoms, defined as mild cognitive impairment due to Alzheimer’s disease or mild dementia. These stages are most relevant because clinical trials of mABs have primarily targeted patients before advanced neurodegeneration occurs. Subgroup analyses will focus on key predefined modifiers: antibody molecule, disease stage (mild cognitive impairment (MCI) due to Alzheimer’s disease versus mild dementia), apolipoprotein E (APOE) ϵ4 status and background symptomatic therapy (acetylcholinesterase inhibitor and/or memantine versus none). Patients with non-Alzheimer’s disease dementias or advanced dementia will be excluded.

Where reported, biomarker confirmation of amyloid pathology (e.g. amyloid PET or cerebrospinal fluid biomarkers) will be extracted and considered in either subgroup analyses or the interpretation of clinical applicability and heterogeneity.

### Types of interventions

The interventions of interest are anti-amyloid mAbs developed for Alzheimer’s disease, irrespective of regulatory approval status, generation or development phase. These include early antibodies targeting soluble or monomeric amyloid-β (e.g. solanezumab, bapineuzumab, crenezumab, gantenerumab, aducanumab) and later-generation antibodies targeting aggregated, protofibrillar or modified amyloid-β species (e.g. lecanemab, donanemab, remternetug).

Only mABs directed against amyloid-β will be included; antibodies targeting tau or other non-amyloid pathways, as well as non-antibody disease-modifying interventions (e.g. small molecules, vaccines, active immunotherapies), will be excluded.

In trials with multiple dose arms or titration schemes, the primary analysis will preferentially include the dose and regimen that most closely reflect the intended clinical use, defined *a priori* as either (a) the dose approved by regulatory authorities, where applicable, (b) the highest tested dose carried forward into phase III trials or (c) the dose identified by trial investigators as the primary or target dose.

If more than one of these criteria apply within a single trial, preference will be given in the order listed above. This hierarchical approach is intended to minimise selective dose choice and between-trial heterogeneity, while ensuring that primary effect estimates reflect doses most relevant to clinical decision-making.

We will include mABs administered via the intravenous or subcutaneous route, consistent with formulations evaluated in RCTs.

#### Comparators

##### Placebo

A placebo is an inactive infusion intended to have no therapeutic or pharmacological effect. Placebo comparators will be included regardless of infusion duration, frequency or appearance.

##### Standard of care

We will include trials in which anti-amyloid mABs are compared against standard-of-care therapy for Alzheimer’s disease, including acetylcholinesterase inhibitors (donepezil, rivastigmine, galantamine) and/or memantine, as appropriate for disease stage.

##### Other active pharmacological interventions

We will include head-to-head or active-comparator trials (if available) that directly compare different anti-amyloid mABs, or those that evaluate these antibodies against other disease-modifying or amyloid-targeting pharmacological agents.

Because many eligible trials are expected to compare anti-amyloid mABs with placebo in addition to background symptomatic therapy, baseline use of acetylcholinesterase inhibitors and/or memantine will be extracted and considered in subgroup analyses and interpretation where data permit.

### Outcome measures

Outcomes will be assessed as defined in each trial protocol and grouped using standard Alzheimer’s disease research domains. Where possible, outcomes will be standardised across studies using established scales and expressed as common metrics (e.g. change from baseline).

The primary analysis will use outcomes measured closest to the prespecified 18-month time point (approximately 76–80 weeks) after randomisation, reflecting the most common primary end-point timing in pivotal phase III trials of anti-amyloid mABs. When multiple time points are reported, data closest to this time point will be selected.

Additional time points (e.g. approximately 6, 12, 24 or 36 months) will be explored in sensitivity analyses or summarised narratively, as appropriate. Trials without outcome data near the 18-month time point (e.g. shorter phase II studies or trials with extended follow-up beyond 24 months) will be analysed separately at their longest reported follow-up, and included only in prespecified time-stratified or sensitivity analyses, or summarised narratively.

Follow-up stratified sensitivity analyses (e.g. ≤12 versus ≥18 versus ≥24 months) will be conducted when data permit. Although the primary analysis focuses on outcomes closest to the 18-month time point, interpretation will also consider the consistency and direction of effects across available time points, where data permit, to better reflect the longitudinal and potentially disease-modifying nature of these interventions.

Outcomes will be pre-classified by importance using the Grading of Recommendations, Assessment, Development and Evaluation (GRADE) framework. Critical outcomes, which are essential for assessing the overall benefit–risk balance, will form the basis of the Summary of findings table, with important outcomes providing supporting contextual information.

Outcome prioritisation was informed by established core outcome frameworks in Alzheimer’s disease, drawing on both the early consensus proposals by Vellas et al and the more recent Delphi-based core outcome set reported by Ellison et al, which distinguish outcome domains, constructs and measurement instruments.^14,15^ Given the focus on pivotal RCTs informing regulatory decisions, this hierarchy was adapted to prioritise global clinical outcomes commonly used as primary or co-primary endpoints in phase III anti-amyloid trials, even when ranked lower in the Delphi exercise. Within global, functional and cognitive domains, measurement instruments were re-ranked to prioritise those most consistently used and most sensitive to change in pivotal trials; the original Delphi ordering was retained for other domains.

Data extraction and synthesis will follow this predefined hierarchy, with the highest-ranked construct and instrument selected for quantitative synthesis. Lower-ranked outcomes will be included only in sensitivity analyses or summarised narratively. The full hierarchy is presented in Table 1.

**Table 1:** Outcomes and outcome measures for symptomatic Alzheimer’s clinical syndrome, informed by established conceptual frameworks in Alzheimer’s disease clinical research, including early proposals emphasising cognitive outcomes in disease-modifying trials^14^ and the Delphi consensus hierarchy,^15^ and adapted to reflect the classification of critical and important outcomes in the present review, with re-ranking of selected measurement instruments to align with those most consistently used in pivotal phase III anti-amyloid trials Table 1 long description.

Classification	Outcome domains	Constructs	Measurement instruments
Critical outcomes	Global outcomes	Identifying individuals’ needs and wants Global improvement Staging severity of dementia	CDR-SB CGIC/ADCS-CGIC/CIBIC+ CDR (global score) GDS Nutritional status (BMI) – not prioritised
	Cognitive abilities	Memory Executive functions Language and communication Judgement and insight Orientation Spatial cognition	ADAS-Cog MMSE MoCA RAVLT TMT-B ACE-R TMT-A
	Functional ability and dependency	ADLs and IADLs Independence and autonomy Social engagement Cognitive engagement Physical health and mobility	ADCS-ADL A-IADL-Q/A-IADL-Q-SV FAQ (IADL) Lawton IADL Barthel Index Katz ADL
	Safety	Serious adverse events Treatment discontinuation due to adverse effects Amyloid-related imaging abnormalities	Incidence of serious adverse events Discontinuation due to adverse effects ARIA-E and ARIA-H detected on MRI
Important outcomes	Behavioural and neuropsychiatric symptoms	Aggressive, challenging and unpredictable behaviour Agitation Depression Personality changes Apathy Anxiety and insecurity Sleep patterns Mood Hallucinations Wandering Delusions/other psychotic behaviour Disinhibition	NPI NPI-Q GDSa NPI-12 CSDD HDRS DAS
	Patient quality of life (QoL)	Patient QoL Impact on relationships Social contact Remaining active Maintaining ability to participate in hobbies Access to dementia-friendly environments Treatment side-effects Sexual health	QoL-AD DEMQoL and DEMQoL-Proxy EQ-5D-5L EQ-5D-3L WHOQoL
	QoL of caregivers and families	Caregiver support Overall impact on caregiver Caregiver/family mental and physical health Caregiver self-efficacy Relationship between caregiver and patient Family involvement in care Other caregiver commitments/loss of free time Spouse’s ‘duty’ to care	ZBI CarerQoL-7D NPI-D CAS GHQ HDRS CES-D
	Health, social care and treatment-related outcomes	Access and use of health services and disease information Delaying entry into institutional care Delirium Falls Hospitalisation Assessment of decision to treat disease-related symptoms Stability of symptoms Frailty Medication side-effects Frequent infections Malnutrition Dysphagia Time to mortality	Direct non-medical costs Long-term institutional care costs Hospital in-patient costs Resource use inventory Accident and emergency costs Prescriptions Hospital out-patient costs Out-of-pocket costs Cost to caregiver/family in terms of ‘time out of role’/time spent caring

Global outcomes: CDR-SB, Clinical Dementia Rating–Sum of Boxes; CGIC, Clinical Global Impression of Change; ADCS-CGIC, Alzheimer’s Disease Cooperative Study–Clinical Global Impression of Change; CIBIC+, Clinician’s Interview-Based Impression of Change Plus Caregiver Input; CDR, Clinical Dementia Rating; GDS, Global Deterioration Scale; BMI, body mass index.

Cognitive outcomes: ADAS-Cog, Alzheimer’s Disease Assessment Scale–Cognitive Subscale; MMSE, Mini-Mental State Examination; MoCA, Montreal Cognitive Assessment; RAVLT, Rey Auditory Verbal Learning Test; TMT-B, Trail Making Test Part B; ACE-R, Addenbrooke’s Cognitive Examination–Revised; TMT-A, Trail Making Test Part A.

Functional outcomes: ADLs, Activities of Daily Living; IADLs, Instrumental Activities of Daily Living; ADCS-ADL, Alzheimer’s Disease Cooperative Study–Activities of Daily Living Inventory; A-IADL-Q, Amsterdam Instrumental Activities of Daily Living Questionnaire; A-IADL-Q-SV, Amsterdam Instrumental Activities of Daily Living Questionnaire–Short Version; FAQ, Functional Activities Questionnaire; Lawton IADL, Lawton Instrumental Activities of Daily Living Scale; Katz ADL, Katz Index of Independence in Activities of Daily Living.

Safety outcomes: ARIA-E, amyloid-related imaging abnormality–oedema; ARIA-H, amyloid-related imaging abnormality–haemorrhage; MRI, magnetic resonance imaging.

Neuropsychiatric outcomes: NPI, Neuropsychiatric Inventory; NPI-Q, Neuropsychiatric Inventory–Questionnaire; GDSa, Geriatric Depression Scale (abbreviated form); NPI-12, Neuropsychiatric Inventory (12-item version); CSDD, Cornell Scale for Depression in Dementia; HDRS, Hamilton Depression Rating Scale; DAS, Dementia Apathy Scale.

Quality of life (QoL) outcomes: QoL-AD, Quality of Life in Alzheimer’s Disease; DEMQoL, Dementia Quality of Life questionnaire; DEMQoL-Proxy, proxy version of the Dementia Quality of Life questionnaire; EQ-5D-5L, EuroQol 5-Dimension questionnaire (5-level version); EQ-5D-3L, EuroQol 5-Dimension questionnaire (3-level version); WHOQoL, World Health Organization Quality of Life assessment.

Caregiver outcomes: ZBI, Zarit Burden Interview; CarerQoL-7D, Care-Related Quality of Life instrument (7-dimension version); NPI-D, Neuropsychiatric Inventory Caregiver Distress Scale; CAS, Caregiver Assessment Scale; GHQ, General Health Questionnaire; CES-D, Center for Epidemiologic Studies Depression Scale.

#### Critical outcomes

- Global clinical change and disease severity;
- cognitive abilities;
- functional ability and dependency;
- safety (composite domain).

The last of these includes the following: (a) incidence of serious adverse events, (b) treatment discontinuation due to adverse effects and (c) amyloid-related imaging abnormalities (ARIA-E and ARIA-H, relating to cerebral oedema and haemorrhages, respectively) detected on MRI: ARIA outcomes will be further characterised by symptom status (symptomatic versus asymptomatic), severity grading and ARIA-H subtypes (microhaemorrhages and superficial siderosis), where reported.

Each adverse effect will be analysed and reported separately, following the guidance of the Cochrane Handbook (chapter 19.5^16^), to avoid obscuring genuine differences between specific harms. Although safety outcomes are conceptually grouped under a single domain, all three components will be presented as separate outcomes due to their independent clinical significance. This approach ensures that key safety dimensions – systemic toxicity, treatment tolerability and ARIA – are transparently represented in the Summary of findings tables and analyses.

ARIA outcomes are expected to occur predominantly during the early treatment period, particularly during dose-escalation phases. Therefore, in addition to analyses at the primary time point, ARIA events will be interpreted with attention given to their temporal distribution, including earlier time points where available.

#### Important outcomes

- Behavioural and neuropsychiatric symptoms;
- quality of life (QoL) of patients;
- QoL of caregivers and family members;
- health-, social care- and treatment-related outcomes.

Health-, social care- and treatment-related outcomes will be extracted and synthesised using a prespecified framework. Given the heterogeneity and incomplete reporting of these outcomes across RCTs, we anticipate that quantitative synthesis will rarely be appropriate. These outcomes will therefore be synthesised primarily using a structured narrative approach, following the synthesis without meta-analysis (SWiM) guideline where applicable.

Extracted data will include, where reported, treatment delivery requirements (e.g. infusion frequency and duration), monitoring and diagnostic infrastructure (e.g. MRI frequency for ARIA surveillance), healthcare utilisation (e.g. hospitalisation, specialist visits) and trial-reported cost or resource-use components. No formal cost-effectiveness or pooled economic analyses will be conducted based on randomised evidence alone.

### Search methods for identification of studies

A comprehensive search strategy will be developed and tailored for each database to identify all relevant trials for inclusion in this review. The core strategy will be designed for Ovid MEDLINE, then adapted to account for variations in controlled vocabulary and search syntax across databases (see Supplementary Material 1).

#### Electronic searches

The following databases will be systematically searched:

- Ovid MEDLINE (from 1946 to present);
- Embase (Elsevier, Embase.com) (from 1947 to present);
- CENTRAL, via The Cochrane Library (from inception).

#### Searching other resources

We will screen the reference lists of all included studies and relevant systematic reviews to identify additional eligible trials. To ensure completeness, we will review retraction notices, errata and expressions of concern linked to any included or potentially eligible studies.

Study authors and pharmaceutical companies will be contacted for missing or unpublished data, or for details of ongoing or completed trials that have not yet been reported.

We will also search the following trial registries for relevant ongoing or unpublished studies:

- ClinicalTrials.gov;
- WHO International Clinical Trials Registry Platform (from inception).

When appropriate, subject-matter experts will be consulted to identify unpublished or ongoing trials. To detect retractions or related editorial notices, we will examine the Retraction Watch database and the websites of journals in which included trials were published.

### Data collection and analysis

#### Selection of studies

Two reviewers from among K.M., F.F., Y.G., S.H., S.S., M.N., R.M., K.I., H.K., T.T., Y.I. and I.A. will independently screen all titles and abstracts yielded from the search strategy to identify potentially eligible trials. Full-text versions of any records deemed relevant will then be retrieved and evaluated against the predefined inclusion criteria. Any discrepancies in judgement between the two reviewers will be discussed with a third author (S.K.) until consensus is achieved.

All studies excluded following full-text review will be listed in a ‘Characteristics of excluded studies’ table, with explicit documentation of the reasons for exclusion. In cases where unexpected methodological or clinical issues emerge during screening, any *post hoc* decisions regarding inclusion or exclusion will be treated as protocol deviations or amendments, dated, justified and reported transparently in the final review documentation.

#### Data extraction and management

Data extraction will be performed independently by pairs of reviewers using a piloted template in Covidence, a web-based systematic review management platform (Veritas Health Innovation, Melbourne, Australia; https://www.covidence.org). One reviewer (S.K.) will extract data from all included studies, with a second reviewer drawn from the following pool: H.O., Sohail B., Sahil B., R.O., R.M. and I.A. Discrepancies between reviewers will be resolved through discussion. Any additional information judged to be relevant to the review objectives will also be captured. For reports published in languages other than English, translation support will be obtained as needed.

Prior to formal data collection, the data extraction form will be piloted to ensure clarity and consistency across reviewers.

#### Risk of bias assessment in included studies

The methodological quality of included randomised trials will be evaluated using the Cochrane risk of bias 2 (RoB 2) tool.

This assessment will address the following domains:

- bias arising from the randomisation process;
- bias due to deviations from intended interventions;
- bias due to missing outcome data;
- bias in measurement of outcomes;
- bias in selection of the reported results.

Risk of bias will be assessed independently by pairs of reviewers using the RoB 2 tool. One reviewer (S.K.) will assess risk of bias for all included studies, with a second reviewer drawn from the following pool: H.O., Sohail B., Sahil B., R.O., Y.I. and I.A. Discrepancies in judgements will be resolved through discussion. Judgements will be categorised as either low risk, some concerns or high risk, in accordance with the RoB 2 decision algorithms based on the signalling questions. The RoB 2 Excel tool (22 August 2019 release; RoB 2 Development Group/Cochrane; London, UK; https://www.riskofbias.info/welcome/rob-2-0-tool/current-version-of-rob-2) will be used for data management.^17^

A graphical summary will illustrate the distribution of studies across risk categories, and a detailed narrative assessment will be presented in the ‘Characteristics of included studies’ table. If clarifications or additional details are required, corresponding or co-authors will be contacted by email. The overall risk of bias for each study will be classified as either low (all domains low risk), some concerns (≥1 domain with some concerns and none high) or high (≥1 domain high risk).

#### Measures of treatment effect

For dichotomous outcomes, we will calculate risk ratios with 95% confidence intervals. For continuous outcomes measured on the same scale, we will calculate mean differences and their 95% confidence intervals. When studies employ different scales to assess comparable outcomes, standardised mean differences will be used.

When standardised mean differences are used, we will contextualise these where feasible by either back-translating them into commonly used scales or interpreting them using representative standard deviations from widely used Alzheimer’s disease measures.

For recurrent or count outcomes, treatment effects will be summarised using rate ratios, following section 6.7.1 of the Cochrane Handbook for Systematic Reviews of Interventions, version 6.4 (updated August 2023^16^).

For rare dichotomous outcomes, including infrequent safety events, a continuity correction of 0.5 will be applied for single-zero studies. Double-zero studies will be excluded from primary meta-analyses of relative effects because they do not contribute information to effect estimation, but they will be documented and considered in sensitivity analyses where appropriate. Alternative methods for rare events, such as Peto odds ratios, may be explored in sensitivity analyses when appropriate.

#### Unit of analysis issues

In multi-arm trials with more than two relevant intervention groups, the number of participants in the shared control arm will be divided proportionally across comparisons to avoid double-counting.

The primary time point for analysis will be described in section 4 (Outcome measures). Briefly, outcomes measured closest to the 12- to 18-month window following randomisation will be prioritised to maximise comparability across studies. Trials with shorter follow-up will be analysed separately at their longest available time point and incorporated only into prespecified time-stratified or sensitivity analyses, or summarised narratively, as appropriate.

Participants will be treated as the unit of analysis in all cases. For outcomes involving recurrent or repeated events, rate ratios will be calculated as described above.

#### Dealing with missing data

When essential outcome data are missing, we will attempt to obtain these by contacting the corresponding authors of the relevant studies. Analyses will be conducted using the intention-to-treat principle whenever possible.

For continuous outcomes, when standard deviations are not reported we will derive these from available statistics (e.g. standard errors, confidence intervals, *p*-values or *t*-statistics) using standard Cochrane methods.^16^ If these cannot be obtained, we will impute standard deviations using the pooled standard deviation from other included studies reporting the same outcome at the same time point.

When both change-from-baseline and end-point values are available for the same outcome, we will preferentially use change-from-baseline data, because these are generally more efficient and account for baseline imbalance; if change scores are unavailable, end-point values will be used.

Change-from-baseline and end-point values may be pooled in the same meta-analysis when they measure the same outcome on the same scale.

For trials with substantial missing outcome data due to attrition, we will assess the risk of bias arising from missing data using the RoB 2 tool.^17^ We will perform sensitivity analyses excluding studies judged to be at high risk of bias due to missing outcome data and, where appropriate, explore the impact of alternative assumptions about missing data. These prespecified approaches are intended to reduce analytic flexibility, ensure consistency across trials with heterogeneous reporting and improve the robustness and interpretability of effect estimates in the presence of missing data.

The robustness of the findings to assumptions about missing data will be examined in sensitivity analyses, and the potential influence of missing data on the overall conclusions will be discussed.

#### Reporting bias assessment

Potential publication bias will be evaluated following the recommendations outlined in section 13.3.5 of the Cochrane Handbook for Systematic Reviews of Interventions.^16^

Funnel plots will be constructed only when at least ten studies are available for a given outcome, and asymmetry will be interpreted cautiously given the limited power of such methods in small meta-analyses. Formal statistical tests for funnel plot asymmetry (e.g. Egger’s test) will be performed only when at least ten studies contribute to the analysis, and will be considered exploratory.

Trials identified through trial registries (e.g. ClinicalTrials.gov) with posted results but no full journal publication will be included if sufficient outcome data are available for extraction. These trials will be assessed for risk of bias using the same criteria as fully published studies, and clearly identified as registry-only reports. Sensitivity analyses excluding registry-only trials will be conducted to assess their influence on pooled estimates, where appropriate.

#### Synthesis methods

We will perform meta-analyses using a random-effects model with a restricted maximum-likelihood estimator for between-study variance, as recommended for improved accuracy.^18^ Wald-type confidence intervals will be used by default for pooled estimates. When the number of studies is small and between-study variance is greater than zero, we will adopt the Hartung–Knapp–Sidik–Jonkman method, consistent with section 10.10.4.4 of the Cochrane Handbook.^16^

The primary unit of quantitative synthesis will be the individual antibody molecule. Meta-analyses will therefore be conducted separately for each anti-amyloid mAB.

This prespecification is justified by well-recognised biological and trial-design heterogeneity across anti-amyloid mABs, including differences in target engagement, epitope specificity, dosing strategies and safety profiles, which limit the interpretability of a class-wide pooled estimate.

For antibody molecules represented by a single eligible study, results will be presented descriptively without quantitative pooling. Any pooling across different antibody molecules will not be considered a primary analysis. To support class-level interpretation without implying a class-wide pooled effect estimate, we will also provide a structured cross-molecule comparative synthesis through narrative summaries and evidence-mapping, including target profile, development stage, regulatory status and key efficacy and safety features where applicable.

Meta-analyses will be undertaken only when appropriate, based on data availability and the degree of statistical heterogeneity. Analyses will be performed using RevMan Web, version 2.2.1, a browser-based systematic review and meta-analysis platform (Cochrane, London, UK; https://revman.cochrane.org), with forest plots generated to visually display effect sizes.^19^ If quantitative synthesis is not feasible, findings will be presented according to the SWiM guideline.^20^

For health system-, social care- and resource-related outcomes, synthesis will be primarily narrative. Quantitative pooling will not be attempted unless outcomes are reported using sufficiently comparable definitions across multiple trials, which is considered unlikely. Narrative synthesis will focus on direction, consistency and key implementation-relevant features rather than on effect size estimation.

#### Investigation of heterogeneity and subgroup analysis

We will assess heterogeneity by verifying data accuracy, visually inspecting forest plots and examining the influence of individual studies. If removal of any study contributing ≤10% of the total weight materially alters the pooled estimate, this will be reported as a sensitivity analysis. In the presence of substantial heterogeneity – particularly when effect estimates differ in direction – we will refrain from pooling and instead provide a narrative synthesis.

All subgroup analyses will be considered exploratory and interpreted cautiously.

Subgroup analyses may be conducted for critical outcomes based on the following factors:

- antibody molecule;
- disease stage (MCI due to Alzheimer’s disease versus mild dementia);
- APOE ϵ4 status (carriers versus non-carriers);
- background symptomatic therapy (acetylcholinesterase inhibitors and/or memantine versus none).

Subgroup comparisons will primarily use trial-level subgroup estimates. Within-study subgroup estimates will be considered only when the subgroup factor was used for stratified randomisation and a prespecified test for interaction was reported; such results will not be pooled with trial-level estimates, to avoid double-counting. Subgroup differences will be assessed using the formal test implemented in RevMan Web.

Meta-regression will be considered only when ten or more studies are available for a given outcome, and results will be interpreted cautiously given the observational nature of these analyses.

#### Equity-related assessment

We will report participant characteristics according to the PROGRESS-Plus framework, including place of residence, race/ethnicity/culture/language, occupation, gender/sex, religion, education, socioeconomic status, social capital, age, sexual orientation and disability. In addition, we will examine whether the effects of anti-amyloid mABs differ across these subgroups and whether such variations may contribute to health inequities. Particular attention will be given to age, sex, APOE ϵ4 carrier status, education level and socioeconomic status, because these factors may influence access to diagnosis, eligibility for treatment, adherence and treatment response. We will summarise the representation of disadvantaged or under-studied populations (e.g. individuals from low- and middle-income countries or those with limited healthcare access) and assess whether existing evidence adequately reflects these groups.

#### Sensitivity analysis

We will conduct sensitivity analyses for the prespecified critical outcomes – global clinical change and disease severity, cognitive abilities, functional ability and dependency, and safety (including ARIA events) – to assess the robustness of our conclusions under alternative analytical assumptions. These analyses will include the following: (a) inclusion of all eligible dose arms, where feasible, to examine the impact of dose selection and to explore potential dose–response relationships; (b) exclusion of studies assessed as having a high risk of bias; and (c) evaluation of the influence of missing outcome data or imputed values.

Additional sensitivity analyses will assess the impact of key methodological choices, including the use of fixed-effect versus random-effects models and the exclusion of open-label extension data. If alternative analytical decisions materially alter the direction or magnitude of effect estimates, these findings will be reported narratively and summarised in sensitivity tables.

#### Certainty of the evidence assessment

The certainty of the body of evidence will be evaluated using the GRADE methodology, and the results will be summarised in a Summary of findings table.^21^ In accordance with the GRADE framework, evidence from RCTs will initially be considered high certainty unless downgraded due to concerns in one or more of the following domains: risk of bias, inconsistency, indirectness, imprecision or publication bias. Depending on the extent and seriousness of these limitations, the certainty rating may be reduced by either one level (moderate), two levels (low) or three levels (very low).^21^

Two reviewers (S.K., H.O.) will independently perform the GRADE assessments, with any differences in judgement resolved through discussion or arbitration by a third reviewer (N.W.).

A Summary of findings table will present anti-amyloid mAbs versus placebo or standard care for the prespecified critical outcomes: global clinical change and disease severity; cognitive abilities; functional ability and dependency; and three separate safety outcomes: serious adverse events, treatment discontinuation due to adverse effects, and ARIA-E/H. Important outcomes, including neuropsychiatric symptoms, quality of life and health-, social care- and treatment-related outcomes, will be summarised alongside where appropriate.

### Living systematic review process

This systematic review will be conducted and reported in accordance with the Cochrane Handbook for Systematic Reviews of Interventions, PRISMA 2020 and PRISMA-LSR 2024.^22,23^

#### Living mode parameters

This review is designed and maintained as a living systematic review. Electronic database searches will be updated at prespecified 6-month intervals. In addition, unscheduled updates may be triggered by major regulatory approvals or the publication of pivotal RCTs relevant to the interventions of interest.

Following each search update, newly identified records will undergo the same two-stage screening process (title/abstract screening followed by full-text review) by two independent reviewers. Data extraction, risk of bias assessment and certainty of evidence assessment will be performed for newly eligible studies using the same prespecified methods applied in the initial review.

Meta-analyses, subgroup analyses, sensitivity analyses and GRADE assessments will be re-run after each update when new eligible evidence is identified. No analytical methods will be applied solely because of the living mode, other than repeated application of the prespecified synthesis methods to the updated evidence base.

#### Versioning, dissemination and publication

Each update of the review will be released as a new version, with a unique version number and date. Updated versions will be made publicly available through an online repository, with clear documentation of changes in included studies, effect estimates and certainty of evidence compared with prior versions.

A formal journal update will be considered when newly incorporated evidence results in either (a) a change in the direction or magnitude of effect estimates, (b) a change in the certainty of evidence, (c) a modification of clinical conclusions or (d) the emergence of newly approved interventions within the scope of the review.

#### Updates to previously included studies

Data, risk of bias assessments and outcome information for previously included studies will be updated when new or revised information becomes available, including longer follow-up data or corrected trial reports. Any such updates will be documented and applied consistently across all versions of the review.

#### Retirement from the living mode

The review will be retired from the living mode if no new eligible studies are identified across two consecutive scheduled search updates, or if the intervention landscape becomes stable with no anticipated future randomised evidence. The rationale for retirement will be explicitly reported in the final living version.

## Discussion

Monoclonal antibodies in the treatment of Alzheimer’s disease represent a major scientific milestone and, at the same time, one of the most controversial developments in contemporary neurology. These therapies offer the first plausible disease-modifying approach for Alzheimer’s disease; however, their accelerated regulatory approvals have intensified debate regarding the appropriate balance among innovation, evidentiary standards and clinical relevance. The central unresolved question remains whether reductions in cerebral amyloid burden translate into clinically meaningful and durable benefits in cognition, functional ability and independence.

For patients and families, anti-amyloid mAB therapies embody both promise and dilemma – potential slowing of disease progression weighed against substantial treatment burdens, including regular infusions, repeated MRI monitoring and the risk of ARIA. For clinicians, a clear understanding of the magnitude and certainty of benefit, alongside a transparent appraisal of harms, is essential to support shared decision-making in the context of modest effect sizes and heterogeneous treatment responses.

At the health system and policy level, anti-amyloid therapies have the potential to reshape the organisation of Alzheimer’s disease care. High acquisition costs, specialised infusion infrastructure, biomarker-based diagnostic pathways and intensive safety monitoring requirements carry important implications for healthcare delivery, resource allocation and equity of access. These considerations extend beyond drug efficacy alone and intersect with broader questions of who is eligible for treatment, under what conditions and within which health system constraints.

The limitations of this review primarily reflect the constraints of the underlying randomised evidence. Although RCTs provide robust estimates of efficacy and safety, they are not designed to capture broader health system dimensions such as service capacity, workforce requirements, long-term sustainability or downstream costs. In addition, health system and resource use outcomes are inconsistently defined and variably reported across trials. In this review, ‘policy implications’ therefore refer specifically to implementation-relevant factors that are directly reported in, or cautiously inferable from, trial data, such as treatment delivery requirements, monitoring of burden and healthcare utilisation. Consequently, policy and system-level conclusions will be interpreted cautiously and will be based primarily on trial-reported evidence, with these outcomes synthesised mainly through structured narrative approaches.

By maintaining this work as a living systematic review, emerging randomised evidence can be rapidly incorporated as new mABs are developed, trials mature or regulatory decisions evolve. This approach allows conclusions to be refined over time while preserving methodological consistency and transparency. By explicitly integrating both the strengths and the inherent limitations of randomised evidence, this review aims to provide clinicians, policy-makers and payers with a rigorous, current and appropriately qualified synthesis of the benefits, harms and policy-relevant considerations of disease-modifying therapy for Alzheimer’s disease.

## Supporting information

10.1192/bjo.2026.12044.sm001 Kim et al. supplementary material Kim et al. supplementary material

## Data Availability

Data availability is not applicable to this article because no new data were created or analysed in this study.

## Supplementary material

The supplementary material is available online at https://doi.org/10.1192/bjo.2026.12044

## Table 1 Long description

A table categorizing outcomes and measurement instruments for symptomatic Alzheimer’s clinical syndrome. The table is divided into several rows and columns, with the columns labeled Classification, Outcome domains, Constructs, and Measurement instruments. The table includes critical outcomes and important outcomes, each with various subcategories. Critical outcomes include Global outcomes, Cognitive abilities, Functional ability and dependency, and Safety. Important outcomes include Behavioural and neuropsychiatric symptoms, Patient quality of life (QoL), QoL of caregivers and families, and Health, social care and treatment-related outcomes. Each row lists specific constructs and corresponding measurement instruments. The table provides a detailed hierarchy for data extraction and synthesis in clinical research.

Navigate back to Table 1.
