# Supplementary material for: Efficacy, safety and policy implications of anti-amyloid monoclonal antibodies for Alzheimer’s disease: protocol for a living systematic review and meta-analysis
Source: BJPsych Open. 2026 Jul 9;12(4):e182. doi: 10.1192/bjo.2026.12044 (PMC13359045; doi:10.1192/bjo.2026.12044)
Supplement: Kim et al. supplementary material [file S2056472426120444sup001.docx]

**Supplemental Material 1**

**Search Strategies**

**Ovid MEDLINE (11/7/2025)**

1. exp Monoclonal Antibodies/

2. ((monoclonal adj2 antibod*) or mAb or mAbs or “anti-amyloid” or “anti amyloid” or “anti-Aβ” or “anti Abeta” or “anti amyloid-beta”).ti,ab.

3. 1 or 2

4. exp Alzheimer Disease/

5. ((Alzheimer* adj2 (disease* or dementia*)) or AD or “early Alzheimer*” or “mild cognitive impairment” or MCI).ti,ab.

6. 4 or 5

7. 3 and 6

8. exp randomized controlled trial/

9. controlled clinical [trial.pt](http://trial.pt).

10. randomized.ab.

11. placebo.ab.

12. drug therapy.fs.

13. randomly.ab.

14. trial.ab.

15. groups.ab.

16. 8 or 9 or 10 or 11 or 12 or 13 or 14 or 15

17. exp animals/ not humans.sh.

18. 16 not 17

19. 7 and 18

**Embase (Elsevier, Embase.com) (11/7/2025)**

#1. monoclonal antibody'/exp

#2. (monoclonal NEAR/2 antibod* OR mAb OR mAbs OR "anti-amyloid" OR "anti amyloid" OR "anti-Aβ" OR "anti Abeta" OR "anti amyloid-beta"):ti,ab

#3. #1 OR #2

#4. alzheimer disease'/exp

#5. ((Alzheimer* NEAR/2 (disease* OR dementia*)) OR AD OR "early Alzheimer*" OR "mild cognitive impairment" OR MCI):ti,ab

#6. #4 OR #5

#7. #3 AND #6

#8. randomized controlled trial'/exp

#9. controlled clinical trial'/de

#10. randomized:ab

#11. placebo:ab

#12. drug therapy'/exp

#13. randomly:ab

#14. trial:ab

#15. groups:ab

#16. #8 OR #9 OR #10 OR #11 OR #12 OR #13 OR #14 OR #15

#17. animal'/exp NOT 'human'/exp

#18. #16 NOT #17

#19. #7 AND #18

**CENTRAL (11/7/2025)**

#1. [mh "Monoclonal Antibodies"]

#2. ((antibody* NEAR/3 amyloid*) OR "anti amyloid" OR "anti amyloid-beta" OR mAb OR mAbs):ti,ab,kw

#3. #1 or #2

#4. [mh "Alzheimer Disease"] OR ((Alzheimer* NEAR/2 (disease* OR dementia*)) OR AD OR (early NEXT Alzheimer*) OR (mild NEXT cognitive NEXT impairment) OR MCI):ti,ab,kw

#5. #3 and #4
